# Cancer testis antigen Cyclin A1 harbors several HLA-A*02:01-restricted T cell epitopes, which are presented and recognized in vivo

**DOI:** 10.1007/s00262-020-02519-6

**Published:** 2020-03-10

**Authors:** Anja Tatjana Teck, Sabrina Urban, Petra Quass, Annika Nelde, Heiko Schuster, Anne Letsch, Antonia Busse, Juliane Sarah Walz, Ulrich Keilholz, Sebastian Ochsenreither

**Affiliations:** 1 Department of Hematology and Oncology, Campus Benjamin Franklin, Charité Berlin, Berlin, Germany; 2 Charité Comprehensive Cancer Center, Charitéplatz 1, 10117 Berlin, Germany; 3 Department of Immunology, Interfaculty Institute of Cell Biology, University of Tübingen, Tübingen, Germany; 4 Clinical Collaboration Unit Translational Immunology, German Cancer Consortium (DKTK), University Hospital Tübingen, Tübingen, Germany; 5 Immatics Biotechnologies GmbH, Tübingen, Germany; 6 Department of Hematology and Oncology, University of Tübingen, Tübingen, Germany; 7 German Cancer Research Center (DKFZ), Heidelberg, Germany

**Keywords:** Cyclin A1, Epitope, CD8^**+**^ T cell, HLA immunopeptidome, Acute myeloid leukemia, Ovarian carcinoma

## Abstract

Cyclin A1 is a promising antigen for T cell therapy being selectively expressed in high-grade ovarian cancer (OC) and acute myeloid leukemia (AML) stem cells. For adoptive T cell therapy, a single epitope has to be selected, with high affinity to MHC class I and adequate processing and presentation by malignant cells to trigger full activation of specific T cells. In silico prediction with three algorithms indicated 13 peptides of Cyclin A1 9 to 11 amino acids of length to have high affinity to HLA-A*02:01. Ten of them proved to be affine in an HLA stabilization assay using TAP-deficient T2 cells. Their immunogenicity was assessed by repetitive stimulation of CD8^**+**^ T cells from two healthy donors with single-peptide-pulsed dendritic cells or monocytes. Intracellular cytokine staining quantified the enrichment of peptide-specific functional T cells. Seven peptides were immunogenic, three of them against both donors. Specific cell lines were cloned and used in killing assays to demonstrate recognition of endogenous Cyclin A1 in the HLA-A*02:01-positive AML cell line THP-1. Immunopeptidome analysis based on direct isolation of HLA-presented peptides by mass spectrometry of primary AML and OC samples identified four naturally presented epitopes of Cyclin A1. The immunopeptidome of HeLa cells transfected with Cyclin A1 and HLA-A*02:01 revealed six Cyclin A1-derived HLA ligands. Epitope p410–420 showed high affinity to HLA-A*02:01 and immunogenicity in both donors. It proved to be naturally presented on primary AML blast and provoked spontaneous functional response of T cells from treatment naïve OC and, therefore, warrants further development for clinical application.

## Introduction

Immunotherapy has been considered a revolution in the treatment of malignant diseases. While the unspecific approach of immune checkpoint inhibition with inhibition of the interaction of programmed cell death 1 (PD-1) and its ligand PD-L1 has entered clinical routine in many indications [1, 2], targeted T cell therapy too has developed substantially within the last years [3, 4]. Many translational scientists working on targeted T cell therapy have turned from vaccination based on peptides or dendritic cells to adoptive T cell therapy (ACT) approaches using autologous T cells transfected with high-affinity T cell receptors (TCR) against epitopes of tumor-associated antigens (TAA) in context of MHC class I molecules. ACT based on the transfection of patient T cells with a second high-affinity TAA-specific TCR is usually designed with an initial apheresis step followed by isolation, activation, and transfection of a predefined T cell subset. While the T cell product is expanded in vitro, the patient receives lymphodepletion by cytotoxic agents. Then a defined number of transfected autologous T cells are transferred back into the patient, sometimes followed by application of IL2 to prolong persistence of the transfected T cells [5, 6].

Several elements of this complex therapeutic strategy—including TCR isolation and modification, transfection vector design for maximum TCR expression and minimization of TCR chain mispairing, lymphodepletion therapy, and the selection and cultivation of patient T cells—have already been optimized enhancing the general efficacy of ACT in early clinical trials [7–9]. However, the identification of suitable antigens remains the biggest challenge. With increasing TAA-specific effector function of the transfected T cell product, not only clinical efficacy but also the risk of immunogenic side effects by *on target*/*off tumor* effects increases [10]. Consequently, selective high expression in the malignant cell population is the most critical feature of any TAA [11, 12]. However, the selection of the target epitope of such a TAA restricted to a predefined MHC class I molecule as HLA-A*02:01, which is the most common class I molecule in most ethnic groups including Caucasians, is the next equally essential step for the development of ACT strategies [13, 14]. Not the expression of the TAA in the target cell but the density of a respective TAA epitope bound to the MHC molecule defines the susceptibility of the target cell to a T cell bearing the matching TCR. Besides the TAA expression, this peptide MHC complex (pMHC) density is dependent on (1) the effectiveness of the processing machinery to generate exactly this peptide and (2) its affinity to the binding groove of the presenting MHC [14].

We recently described Cyclin A1 as new gametopoiesis-associated cancer testis antigen, which is selectively expressed in AML including its stem cell compartment and OC [11, 15]. Cyclin A1 is essential for male meiosis I and exclusively expressed in male gametocytes. It is not essential for function of physiological somatic cells given that Cyclin A1 knockout mice develop normally except for infertility of the male animals [16, 17]. Overexpression of Cyclin A1 in murine hematopoietic stem cells caused myelodysplasia and acute leukemia [18], indicating its oncogenic potential, which makes downregulation, mutation, or deletion of Cyclin A1 as a resistance mechanism to immunological pressure by a targeted T cell therapy unlikely. Cyclin A1-specific T cell clones selectively lysed Cyclin A1-expressing primary AML blasts showing the potential therapeutic capacity of a Cyclin A1-targeted T cell therapy [11].

Until now, we have identified two HLA-A*02:01-restricted epitopes by stimulation of T cells from two HLA-A*02:01-positive donors with a Cyclin A1 peptide library. However, even though T cells against both epitopes could be isolated from both donors, stimulation with a peptide library resulted in predominant T cell populations specific for different HLA-A*02:01 restricted epitopes [11]. Furthermore, in silico prediction revealed many other candidate peptides with high affinity to HLA-A*02:01.

In this study, we systematically mapped Cyclin A1 for additional HLA-A*02:01-restricted epitopes by in silico prediction of potential epitopes. We assessed the affinity of predicted peptides to HLA-A*02:01 by stabilization assays and their immunogenicity by in vitro stimulation. In parallel, we analyzed the naturally presented MHC class I-restricted peptides (HLA ligandome or immunopeptidome) of AML patient samples and screened for Cyclin A1 derived peptides. With this approach, we identified ten peptides with adequate binding to HLA-A*02:01. We describe four new HLA-A*02:01-restricted epitopes as targets for ACT.

## Materials and methods

### In silico prediction of HLA-A*02:01-binding peptides

Three different epitope prediction algorithms (SYFPEITHI, BIMAS, IEDB analysis resource [19–21]) were used to identify peptides of Cyclin A1 *isoform c* (NM_001111046) with high affinity to HLA-A*02:01. All possible 9-mers and 10-mers were ranked by all algorithms. Peptides ranking within the 10 highest 9-mers or 10-mers were selected for further analysis after peptides with complete homology to other human proteins were discarded.

### HLA stabilization assay

Affinities of peptides were tested by HLA stabilization assay using transporter associated with antigen processing (TAP)-deficient T2 cells. T2 cells were incubated overnight with the respective peptides at a final concentration of 100 μg/ml in serum-free RPMI containing 1 μg/ml β_2_-microglobulin (Sigma-Aldrich, St. Louis, MO, USA). Golgi apparatus was blocked with Brefeldin A (Sigma-Aldrich) for additional 4 h. After this, HLA-A/B/C was stained (W6/32-FITC; BD Biosciences, Franklin Lakes NJ, USA), and percentage of HLA high cells per sample was assessed by flow cytometry. All FACS data were acquired using a FACS Canto II (BD Biosciences) and analyzed with FlowJo software (Ashland, OR, USA).

### HLA ligandome analysis

For the identification of Cyclin A1 peptides processed and presented, HeLa cells were transfected with Cyclin A1 *isoform c* and HLA-A*02:01, both cloned into a pcDNA3.1 vector (Qiagen, Hilden, Germany) using lipofectamine (Invitrogen, Carlsbad, CA, USA). Transfected cells were snapfrozen for HLA ligandome analysis.

HLA class I molecules were isolated by standard immunoaffinity purification [22, 23] using the pan-HLA class-I-specific mAb W6/32 (produced in-house, Department of Immunology Tübingen).

HLA ligand extracts were analyzed as described previously [24]. Peptides were separated by nanoflow HPLC using a 50 µm × 25 cm PepMap rapid separation column (Thermo Fisher Scientific, Waltham, MA, USA) and a 2.4—32.0% acetonitrile gradient over the course of 90 min. Eluted peptides were analyzed in an online-coupled LTQ Orbitrap Fusion Lumos mass spectrometer (Thermo Fisher Scientific) using a top-speed collision-induced dissociation fragmentation method.

Data processing was performed as described previously [24, 25]. Briefly, the Proteome Discoverer (v1.3, Thermo Fisher) was used to integrate the search results of the SEQUEST HT search engine (University of Washington) [26] against the human proteome (Swiss-Prot database, release 2013_09, 20,279 reviewed protein sequences) without enzymatic restriction. The false discovery rate was estimated using the Percolator algorithm 2.04 [27] and limited to 5%. Peptide lengths were limited to 8–12 amino acids. Protein grouping was disabled to ensure multiple annotations. HLA class I annotation was performed using SYFPEITHI 1.0 [21] and NetMHCpan 3.0 [28].

### Generation of T cell lines by in vitro stimulation

Immunogenicity of peptides was assessed by the generation of peptide-specific T cell lines by in vitro stimulation of T cells with single-peptide-pulsed autologous derndritic cells (DC) or peripheral blood mononuclear cells (PBMC), which were isolated by density gradient centrifugation (Biocoll L6115, Biochrom, Berlin, Germany) from buffy coats of healthy donors. HLA-A*02:01 typing was performed serologically using BB7.2 monoclonal antibody (BD Biosciences). CTL were immunomagnetically separated from PBMC with anti-CD8 microbeads (Miltenyi Biotec, Teterow, Germany) and frozen. The plastic-adherent fraction of CD8-depleted PBMC were frozen for restimulation or differentiated to DC with IL-4 and GM-CSF as previously described [11]. After 24 h, maturation of the DC was induced by adding TNFα, IL-1β, IL-6, and prostaglandin E_2_.

For primary stimulation, 1 × 10^6^ DC were pulsed with peptide at a final concentration of 10 µg/ml and co-incubated with 6 × 10^6^ CD8^**+**^ T cells in the presence of 30 ng/ml IL-21 (PeproTech, Rocky Hill, NY, USA). At day 3, 12.5 U/ml IL-2 (Novartis Pharma, Nürnberg, Germany), 10 ng/ml IL-7 (Miltenyi Biotec), and 10 ng/ml IL-15 (R&D systems, Minneapolis, MN, USA) were added. During further culture, half of the medium supplemented with IL-2, IL-7, and Il-15 was replaced every other day. After 10 to 14 days, the CTL were either restimulated with DC as described above or restimulated with the peptide-pulsed plastic-adherent fraction of autologous CD8-depleted PBMC in a ratio CTL/PBMC of 1:2 with cytokine feeding starting from day two and increasing the final IL-2 concentration to 25 U/ml. CTL were enriched by a total of four stimulations.

At the end of each restimulation cycle, specificity of T cells was assessed by ICS of IFNγ induced by peptide-pulsed T2 cells as APC in the presence of monensin (BD Biosciences) for 14 h. Cells were stained with anti-CD8-FITC (HIT8a, BD Biosciences), fixed and permeabilized (Cytofix/Cytoperm-Kit, BD Biosciences), and stained with anti-IFNγ-APC (B27, BD Biosciences) for flow cytometry. For the presentation of the data, specific cells were defined as percentage of cytokine-positive CD8^+^ lymphocytes after exposure to the respective Cyclin-A1 peptide minus this percentage after incubation with an irrelevant Wilms tumor (WT1) peptide.

### Generation of T cell clones

T cell clones were generated by limiting dilution of specific T cell lines and further expanded using the rapid expansion protocol by Greenberg et al*.* containing irradiated mixed feeder cells (PBMC and a lymphoblastoid cell line), 30 ng/ml OKT3 (Thermo Fisher Scientific), and 50 IU/ml IL-2 [29]. Limiting dilution was done likewise with additional 5 ng/ml IL-15 and without changing medium. After 15 days, growing clones were tested for specificity using IFNγ ICS.

### Vital FR assay on peptide-specific lysis

Specific lysis of APC by CTL was tested in a Vital Far Red (FR) assay previously described [30]. In brief, CTL were co-incubated in different effector/target (E/T) ratios with a mixture of *5* × *10*^*3*^ target and *5* × *10*^*3*^ control (C) target cells, which were pre-labeled with different ester dyes (specific targets: carboxyfluorescein-diacetat-succinimidyl-ester 10 µM (CFDA-SE, Life technologies, Waltham, MA, USA), control cells: dimethyldodecylamineoxide-succinimidyl-ester 5 µM (DDAO-SE, Cell Trace Far Red, Life technologies)).

The ratio of specific targets per control cells (T:C ratio_effector_) was assessed by flow cytometry after 4 h of co-incubation with CTL. For normalization, the same ratio was assessed without effectors (T:C ratio_norm_). Specific lysis was calculated using the formula

$${\mathrm{Specific\,lysis}} \left[ \% \right] = 100 \times \left( {1 - \frac{{{\mathrm{T:C\,ratio}}_{{{\mathrm{effector}}}} }}{{{\mathrm{T:C\,ratio}}_{{{\mathrm{norm}}}} }}} \right)$$

Three different pairs of APC were used as target and control cells: T2 pulsed with specific peptide versus T2 with irrelevant peptide; THP-1 IFNγ-stimulated versus THP-1 unstimulated both without peptide; THP-1 versus T2 both IFNγ-stimulated, both without peptide.

### Detection of Cyclin A1-specific T cells ex vivo

After informed consent and in accordance to the local ethics committee, PBMC from six HLA-A*02:01-positive treatment naïve OC patients were collected. PBMC from two HLA-A*02:01-positive healthy donors were analyzed as control. PBMC were incubated with single peptides in a concentration of 10 µg/ml in the presence of Brefeldin A overnight. ICS was performed as described above. T cell markers were stained CD3 SK7-PerCP, CD4 RPA-T4-APC-H7 and CD8 SK1-PE and cytokines were stained IFNγ B27-APC, IL2 5344.111-FITC (all antibodies BD Bioscience) and TNFα Mab11-PE-Cy7 (eBioscience, San Diego, CA, USA). For the presentation of the data, percentage of cytokine-positive cells after exposure to an irrelevant HIV peptide was subtracted from the percentage of cells after exposure to the respective Cyclin A1 peptide.

## Results

### Several unique peptides of Cyclin A1 bind HLA-A*02:01

**Fig. 1:**
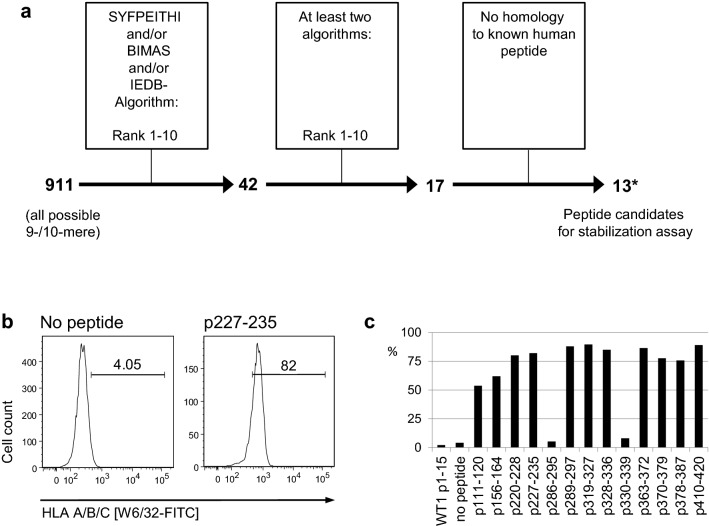
Selection of peptide candidates: **a** In silico prediction and epitope selection, *13 candidates include pre-described epitope p227–235, **b** HLA-A*02:01 stabilization assay, gating on HLA-A*02:01 high cells, positive (p227–235) and negative (no peptide) control, **c** HLA-A*02:01 stabilization assay: percentage of HLA-A*02:01 high cells

For the identification of novel T cell epitopes of Cyclin A1, two strategies were pursued independently. For the first approach, in silico prediction of candidate peptides was performed using three different prediction algorithms. Of all 911 possible 9-mers and 10-mers of Cyclin A1 *isoform c*, 42 peptides were predicted among the ten best HLA-A*02:01 binders by at least one of the three used in silico prediction algorithms. Seventeen peptides ranked among the top 10 binders of at least two algorithms. Four peptides were excluded from further analysis because of perfect homology to other human proteins (Fig. 1a). One of the remaining peptides was the pre-described peptide p227–235, which was used as positive control in the subsequent stabilization assay. As p410–420, one of the corresponding 11-mers was predicted to bind HLA-A*02:01 better than the 10-mer, and the 11-mer was used in further experiments (Table 1).

**Table 1:** Sequences of investigated Cyclin A1 peptide**s**

aa position^**b**^	Sequence	Length (aa)	In silico prediction	HLA stabilization	T cell stimulation tested	HLA ligandome HeLa cells	HLA ligandome primary samples
111–120	GMAFEDVYEV	10	+	+	+		
112–120	MAFEDVYEV	9			−	+	
156–164	SLGTDVINV	9	+	+	+		
220–228	TLYLAVNFL	9	+	+	+		
227–235	FLDRFLSCM^**a**^	9	+	+	+		
286–295	LLLKVLAFDL	10	+	−	−		
289–297	KVLAFDLTV	9	+	+	+		
319–327	NLAKYVAEL	9	+	+	+	+	AML
328–336	SLLEADPFL	9	+	+	+	+	AML
330–339	LEADPFLKYL	10	+	−	−		
341–351	SLIAAAAFCLA^**a**^	11			−		
363–372	TLAAFTGYSL	10	+	+	+		
370–379	YSLSEIVPCL	10	+	+	+		
378–387	CLSELHKAYL	10	+	+	+		
410–418	SLMEPPAVL	9			−	+	
410–419	SLMEPPAVLL	10	+		−	+	AML
410–420	SLMEPPAVLLL	11	+	+	+	+	AML, OC

Sequences of investigated Cyclin A1 peptides, including ^a^two pre-described HLA-A*02:01-restricted epitopes [11]. ^b^Positions are given based on the shorter sequence of isotype c of Cyclin A1

The candidate peptides were then tested in an HLA stabilization assay to confirm the predicted affinity to HLA-A*02:01 in vitro. Two candidates showed results comparable to the negative controls (irrelevant WT1 peptide and no peptide) and were therefore discarded from further analysis (Fig. 1b, c). The remaining 10 peptide candidates were subsequently tested for immunogenicity by in vitro T cell stimulation.

### Cyclin A1 T cell epitopes are naturally presented

In an alternative approach to identify Cyclin A1-derived T cell epitopes, HLA class I peptides were eluted from a Cyclin A1- and HLA-A*02:01-positive cell line to screen for processed and presented peptides of Cyclin A1 *isoform c*. For this, the cervical cancer line HeLa was transfected with HLA-A*02:01 and Cyclin A1. The HLA class I ligandome comprised 1139 unique HLA ligands representing 1209 different source proteins. Six Cyclin A1-derived HLA-A*02:01-restricted peptides were identified (Table 1). All six have already been vetted during the selection process by in silico prediction and HLA stabilization and chosen for further analysis, either as identical peptide (peptides p319–327, p328–336, p410–420) or as longer corresponding peptide to p112–120, p410–418, or p410–419.

Furthermore, a primary sample database of HLA ligandome data from various cancer entities (552 samples with 245 HLA-A*02:01-positive donors) and benign tissue samples (404 samples with 172 HLA-A*02:01-positive donors) was screened for naturally presented Cyclin A1-derived HLA-A*02:01-restricted ligands. Thereby four different Cyclin A1 HLA-A*02:01 ligands could be detected on primary AML and OC samples (Table 1). In particular, the two peptides p328–336 and p410–420 showed high presentation frequencies of 27% (7/26 HLA-A*02:01-positive^**+**^) on primary AML samples. Most importantly, no Cyclin A1-derived peptides were identified on benign tissue samples proving the tumor/leukemia-exclusivity of this antigen even on HLA ligandome level.

### Predicted and in vitro-eluted peptides are immunogenic

**Fig. 2:**
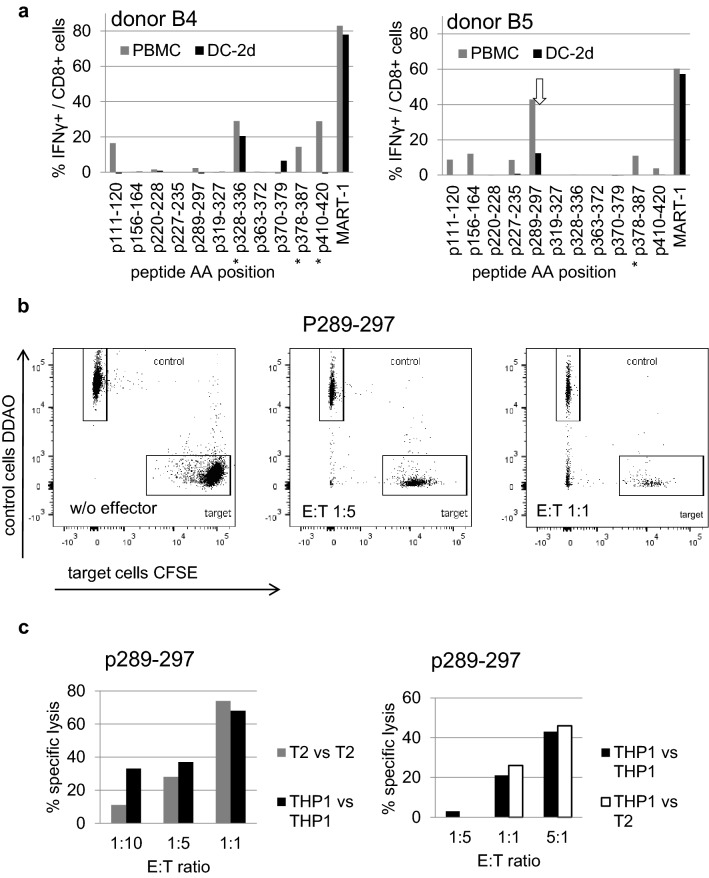
Generation of specific T cell lines in vitro: **a** Highest reached frequencies of specific T cells quantified by IFNγ ICS after three (*) or four stimulations with DC or DC followed by PBMC. The successful clone source is marked by an arrow. **b** Vital FR toxicity assay normalization panel without effector cells (left) and samples with effector CTL (middle, right) against Cyclin A1 p289–297 and target THP-1 cells with and control THP-1 cells without IFNγ exposure. **c** Specific lysis calculated from three different Vital FR settings for target pairs: T2 peptide-pulsed with Cyclin A1 p289–297 as target versus T2 with irrelevant peptide as control (gray), THP1 with and without IFNγ exposure (black) and THP1 versus T2 both with IFNγ exposure (white)

To test whether the 10 peptide candidates of Cyclin A1 are immunogenic, CTL of two healthy donors (B4 and B5) were repetitively stimulated with single-peptide-pulsed autologous cells. The pre-described Cyclin A1 p227–235 and MART-1-peptide ELAGIGILTV served as positive controls. Restimulation was done either with DC or with PBMC. At the end of each cycle, specificity of the lines was assessed by ICS (Fig. 2a).

As already published by us and others, repetitive stimulation with mature DC was less effective for the generation of specific T cell lines than the restimulation with plastic-adherent PBMC [11]. Overall, 12 specific T cell lines against seven different peptides (> 4% IFNγ-positive cells in ICS after stimulation with the respective peptide) could be generated besides five cell lines for the positive controls. Against two peptides, both stimulation protocols resulted in specific lines. Three peptides showed in vitro immunogenicity for both donors: Cyclin A1 p111–120, p378–387, and p410–420. Stimulation with DC accounted for only three specific cell lines. Nevertheless, the only established T cell clone was generated by DC stimulation. The respective line is marked in Fig. 2a.

### CTL specific for Cyclin A1 p289–297 are cytotoxic

Cell lines with sufficient viability after three to four stimulations were exposed to a limiting dilution step to generate specific T cell clones followed by rapid expansion. One clone recognizing Cyclin A1 p289–297 could be established, that specifically lysed APC in Vital FR toxicity assays.

To show specific killing, peptide-pulsed T2 cells were used as APC. T2 pulsed with Cyclin A1 p289–297 served as targets, and T2 pulsed with irrelevant WT1 peptide served as controls. After 4 h of co-incubation with an E/T ratio of 1:1, flow cytometry revealed 74% specific lysis of target cells due to peptide-specific lytic activity of cytotoxic T lymphocytes. With decreasing E/T ratios, the specific killing decreased (Fig. 2c left gray).

To test whether the established CTL recognized endogenous Cyclin A1, THP-1 cells were used as APC. THP-1 are HLA-A*02:01-positive and express low amounts of Cyclin A1 [11]. This expression can be enhanced by incubation with IFNγ. A Vital FR assay was conducted using IFNγ-exposed THP-1 cells as specific and cytokine-naïve THP-1 cells as control cells. Flow cytometry plots illustrate the decrease in viable specific target cells with increasing E/T ratio (Fig. 2b) resulting in a calculational specific lysis at E/T ratio 1:1 of 68% (Fig. 2c left black). To rule out an unspecific effect by the IFNγ treatment of the specific targets, in a third setting IFNγ pretreated T2 cells without peptide-pulse were used as control cells. At a 5:1 E:T ratio, the CTL performed specific lysis of 46% of the THP-1 cells compared to 43% in the THP-1 versus THP-1 setting where the same CTL and the same specific targets were incubated with cytokine-naïve THP-1 as control cells (Fig. 2c right). These cytolytic experiments proved the recognition and therefore adequate processing and presentation of Cyclin A1 epitope p289–297.

### Cyclin A1 epitopes are presented and recognized in vivo

**Fig. 3:**
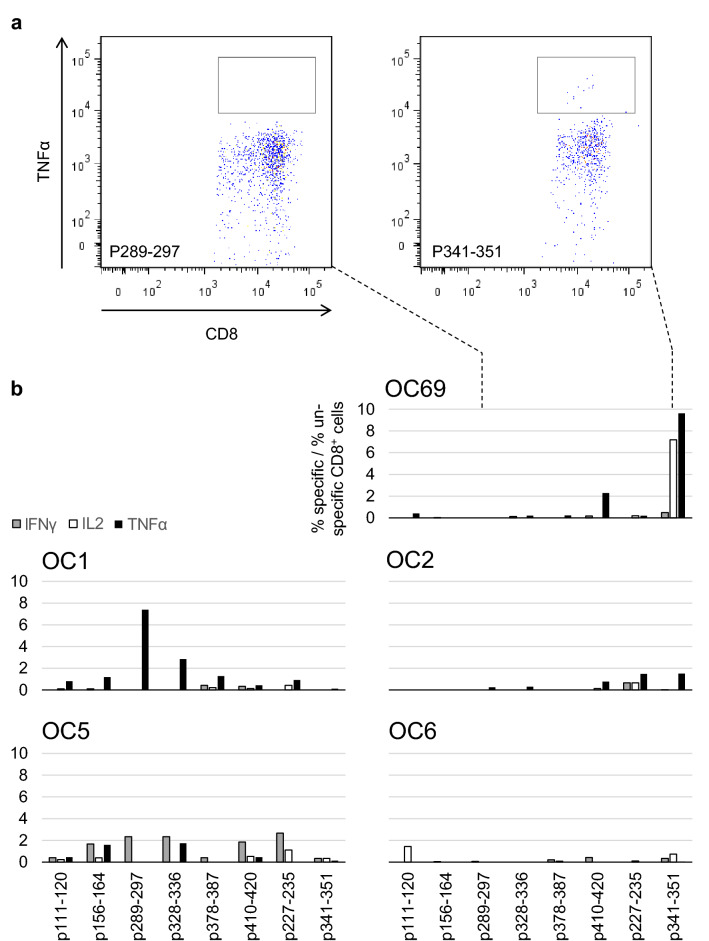
Spontaneous T cell responses against Cyclin A1 epitopes in treatment-naïve OC patients: **a** Example of intracellular TNFα staining of CD8-positive cells of patient OC69. **b** Specific cytokine production of all analyzed patients. Values are given as specific cells per unspecific cells

Spontaneous CTL responses against Cyclin A1 epitopes were assessed in PBMC from six therapy-naïve OC patients and two healthy controls. Intracellular staining of IL2, IFNγ, and TNFα was performed after incubation with the Cyclin A1 peptides or an unspecific HIV peptide (Fig. 3a and not shown). For analysis, the percentage of unspecific T cells was subtracted from the specific response, and the specific response was calculated as the ratio of epitope-specific T cells per unspecific T cells (Fig. 3b). As a selection criterion for epitopes, ‘positive’ was defined as a ratio of more than twofold. The spontaneous responses differed among the patients concerning number of epitopes and produced cytokines. The most and highest responses were TNFα-positive. Patient OC69 showed an IL2/TNFα-positive response seven to nine times as high as the unspecific control. In one of the healthy controls, one TNFα-positive response against p410–420 was detected.

## Discussion

In this study, we utilized several approaches to identify and evaluate HLA-A*02:01-restricted epitopes of the cancer testis antigen Cyclin A1 as target for T cell-based therapies in an as comprehensive way as possible. Ten new candidate peptides were identified by in silico prediction and vetted regarding affinity to HLA-A*02:01, processing and presentation, and immunogenicity both in vitro and in vivo resulting in four new epitopes suitable for further development toward a clinical application.

After the identification of the target antigen, the selection of the T cell epitope is the most crucial step for the development of any targeted TCR-based therapy in oncology. Ultimate criterion for the choice of an epitope is the clinical efficacy of a respective T cell response or a TCR transgenic T cell product. However, several features, which can be assessed in vitro or ex vivo, might predict the clinical usefulness of a given epitope. T cell activation depends mainly on the functional avidity of the effector and the density of the pMHC complexes on the target cell [31]. The former depends on both TCR affinity and density as well as the activation grade of the cell, which is modulated mainly by the micromilieu, co-stimulation, and expressed checkpoint ligands in the tumor compartment, but per se is not dependent on the targeted TAA or epitope [32]. The latter is a function not only of the expression and the degradation rate of the target protein in the target cell, which are both TAA but not epitope-specific, but also on the efficacy of the processing machinery and the affinity of the peptide to the respective MHC molecule [14, 33]. These last two criteria are epitope-specific and therefore the basis for the selection of an epitope as a target for T cell mediated therapy.

Immunodominance of T cell responses against HLA-A*02:01-restricted epitopes has been described for viral antigens, which seems linked to naïve precursor frequencies [34], whereas responses against TAA show substantial inter-individual differences in terms of epitope selectivity and expansion capability. Most likely, this variability is caused by the shaping of the T cell repertoire by negative thymic selection [11, 35]. The in vitro immunogenicity of a self-peptide can be even an indicator for insufficient processing and presentation, which in the thymus would result in circumvention of negative selection of T cells with high-affinity TCR. In contrast to earlier strategies of targeted T cell therapy like vaccination or the application of expanded autologous T cells, modern adoptive T cell therapies are not dependent on the endogenous TCR repertoire of the patient but on the affinity of an exogenous TCR, which can be affinity matured before application [6]. In accordance with these considerations, selection of the targeted epitope should not primarily be based on immunogenicity. Our experiences with the generation of TAA-specific T cell lines against Cyclin A1 and other self-antigens by stimulation both with peptide libraries and single peptides never revealed uniform epitope specificity even though a predominant T cell clone was always detected ([11] and own unpublished data). Baseline immunogenicity at least in some donors of course is a requirement for TCR isolation, which cannot be modeled in a non-humanized system. The generation of T cell clones against an epitope is a useful tool to indirectly prove adequate presentation of the epitope by showing specific killing of tumor cells by the respective clone without peptide pulsing. In case no stimulation steps have been interposed, the detection of spontaneous T cell responses directly ex vivo of course is an indicator for epitope presentation in vivo.

Consequently, direct and indirect indicators for sufficient MHC presentation were used as primary criterion for epitope selection. We postulate that for a suitable epitope its processing and presentation should be proven by a T cell clone recognizing the endogenous TAA in a suitable target cell line. Alternatively, natural presentation should be shown by HLA ligandome analysis, and immunogenicity should be proven in vitro by generating T cell lines and/or in vivo by detection of spontaneous T cell responses. Table 2 summarizes all results collected in this study plus pre-described data on HLA-A*02:01-restricted Cyclin A1 epitopes. According to the above-defined criteria, we now have identified two epitopes, for which recognition of endogenous processed peptide have been shown only by a specific T cell clone (p227–235, p341–351 [11]), three new epitopes with proven presentation and immunogenicity (p111–120, p328–336, p410–420), and one new epitope, which fulfils both criteria (p289–297).

**Table 2:**
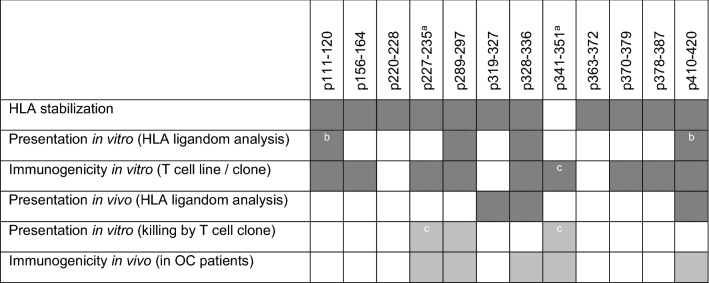
Summary of features of all new and pre-described HLA-A*02:01-restricted Cyclin A1 epitopes

Results of all described criteria for all in silico-predicted epitope candidates. Shaded box equals criterium (left) fulfilled. “Presentation in vitro (killing by T cell clone)” and “immunogenicity in vivo” are shaded lighter to clarify that these criteria are considered secondary as discussed

^a^Pre-described peptides [11]

^b^Whole peptide as well as shorter peptides (p410–419, p410–418)

^c^Pre-described result [11]

This study has limitations, especially regarding the limited number of T cell donors, of T cell lines and clones, and of patient samples for the detection of spontaneous responses. This makes the criterion of a clone spontaneously killing a positive cell line and the criterion of a spontaneous T cell response in patients with Cyclin A1-positive malignancies secondary criteria, which should not primarily be used to select a target epitope based on our data. HLA ligandome analysis has shown high specificity as performed on in vitro transfected cells as well as patient samples. Despite improved detection methods, it still has limited sensitivity dependent on the number of analyzed cells [36], the general HLA surface expression of the respective cells, and the density of presentation of the respective antigen of interest [24, 37]. Therefore, a positive ex vivo detection of a presented epitope is a strong indicator for significant presentation. However, the converse, i.e., no detection by HLA ligandome analysis proving no significant presentation, is not stringent.

In conclusion, we identified four new HLA-A*02:01-restricted epitopes of Cyclin A1 and provide characteristics, which can facilitate the epitope selection for targeted T cell therapy approaches. It is advisable to develop TCR against several of these epitopes, given that the efficacy of a TCR, which is not only a function of the affinity of the TCR to the pMHC but also the efficacy of the processing and presenting of the respective epitope, can only be determined by direct testing.

## Abbreviations

AML: Acute myeloid leukemia
FR: Far red
OC: Ovarian cancer
pMHC: Peptide MHC complex
TAP: Transporter associated with antigen processing
