# Correction to: Cancer testis antigen Cyclin A1 harbors several HLA‑A*02:01‑restricted T cell epitopes, which are presented and recognized in vivo

**DOI:** 10.1007/s00262-021-02977-6

**Published:** 2021-06-25

**Authors:** Anja Tatjana Teck, Sabrina Urban, Petra Quass, Annika Nelde, Heiko Schuster, Anne Letsch, Antonia Busse, Juliane Sarah Walz, Ulrich Keilholz, Sebastian Ochsenreither

**Affiliations:** 1 Department of Hematology and Oncology, Campus Benjamin Franklin, Charité Berlin, Berlin, Germany; 2 Charité Comprehensive Cancer Center, Charitéplatz 1, 10117 Berlin, Germany; 3 Department of Immunology, Interfaculty Institute of Cell Biology, University of Tübingen, Tübingen, Germany; 4 Clinical Collaboration Unit Translational Immunology, German Cancer Consortium (DKTK), University Hospital Tübingen, Tübingen, Germany; 5 Immatics Biotechnologies GmbH, Tübingen, Germany; 6 Department of Hematology and Oncology, University of Tübingen, Tübingen, Germany; 7 German Cancer Research Center (DKFZ), Heidelberg, Germany

## Correction to: Cancer Immunology, Immunotherapy (2020) 69:1217–1227 10.1007/s00262-020-02519-6

The article Cancer testis antigen Cyclin A1 harbors several HLA‑A*02:01‑restricted T cell epitopes, which are presented and recognized in vivo, written by Anja Tatjana Teck, Sabrina Urban, Petra Quass, Annika Nelde, Heiko Schuster, Anne Letsch, Antonia Busse, Juliane Sarah Walz, Ulrich Keilholz and Sebastian Ochsenreither, was originally published Online First without Open Access. After publication in volume 69, issue 7, page 1217–1227 the author decided to opt for Open Choice and to make the article an Open Access publication. Therefore, the copyright of the article has been changed to © The Author(s) 2021 and this article is licensed under a Creative Commons Attribution 4.0 International License, which permits use, sharing, adaptation, distribution and reproduction in any medium or format, as long as you give appropriate credit to the original author(s) and the source, provide a link to the Creative Commons licence, and indicate if changes were made. The images or other third party material in this article are included in the article’s Creative Commons licence, unless indicated otherwise in a credit line to the material. If material is not included in the article’s Creative Commons licence and your intended use is not permitted by statutory regulation or exceeds the permitted use, you will need to obtain permission directly from the copyright holder. To view a copy of this licence, visit http://creativecommons.org/licenses/by/4.0/.

The original article has been corrected.

